# Expression of emotion in music and vocal communication: Introduction to the research topic

**DOI:** 10.3389/fpsyg.2014.00399

**Published:** 2014-05-05

**Authors:** Anjali Bhatara, Petri Laukka, Daniel J. Levitin

**Affiliations:** ^1^Sorbonne Paris Cité, Université Paris DescartesParis, France; ^2^Laboratoire Psychologie de la Perception, CNRS, UMR 8242Paris, France; ^3^Department of Psychology, Stockholm UniversityStockholm, Sweden; ^4^Department of Psychology, McGill UniversityMontreal, QC, Canada

**Keywords:** music, speech, emotion, voice, cross-domain cognition

In social interactions, we must gauge the emotional state of others in order to behave appropriately. We rely heavily on auditory cues, specifically speech prosody, to do this. Music is also a complex auditory signal with the capacity to communicate emotion rapidly and effectively and often occurs in social situations or ceremonies as an emotional unifier.

Scientists and philosophers have speculated about the common cognitive origins of music and language. Perhaps their common origin lies in their efficacy for emotional expression. Unlike semantic or syntactic aspects of language (and music), many of their acoustic and emotional aspects are shared with sounds made by other species (Fitch, 2006); music and speech share a common acoustic code for expressing emotion (Juslin and Laukka, 2003). Until recently, however, scientists working in the two domains of music and speech rarely communicated, so research was restricted to one domain or the other. The purpose of this Research Topic was to bring these researchers together and encourage cross-talk.

Over 25 groups of researchers contributed their expertise, and the included papers give an overview of the diversity of current research, both in terms of research questions and methodology. Some articles focus on aspects in one of the two domains, whereas other articles directly compare, contrast, or combine music and vocal communication.

Empirical studies on music perception include work by Eerola et al. (2013), in which they systematically manipulated musical cues to determine their effects on perception of emotion, and Droit-Volet et al. (2013), who altered acoustic elements associated with emotion to examine the effect of these changes on time perception. Effects of context on music understanding were also investigated: Spreckelmeyer et al. (2013) examined preattentive processing of emotion, measuring ERPs during the processing of a sad tone within the context of happy tones and the reverse. Schellenberg et al. (2012) demonstrated a listener preference for music that expressed emotion contrasting with an established context, and Loui et al. (2013) examined the role of vocals on perceived arousal and valence in songs.

Turning to emotional responses to music, Russo et al. (2013) developed models aimed at predicting the emotion being experienced using information in the listeners' physiological signals, and Altenmüller et al. (2014) used fMRI to investigate the neural basis of episodic memory for arousing film music. Following up on Gabrielsson's (2002) distinction between emotion felt by a listener and emotion expressed by a piece of music, Schubert (2013) provided a review and suggestions for future research on the internal and external loci of musical emotion. There were also two theoretical papers on musical emotions: Flaig and Large (2014) speculated that music may induce affective response by speaking to the brain in its own language by way of neurodynamics, and Allen et al. (2013) presented a view of the general nature of musical emotions based on studies on autism.

In the speech domain, Paulmann et al. (2013) used EEG to investigate influences of arousal and valence on cortical responses to emotional prosody. Rigoulot et al. (2013) used a gating paradigm to demonstrate the importance of utterance-final syllables in emotion recognition. Two papers focused on the role of specific acoustic cues in vocal expression: Weusthoff et al. (2013) discussed the role of fundamental frequency in the success of romantic relationships, and Yanushevskaya et al. (2013) examined the role of loudness, both independently and in conjunction with voice quality.

Several researchers undertook cross-cultural studies of emotion perception in speech and non-verbal vocalizations. Jürgens et al. (2013) examined the perception of German emotional speech tokens across three cultures. Waaramaa and Leisiö (2013) examined the recognition of emotion in Finnish pseudo-sentences by listeners from five countries. There were also three cross-cultural investigations of non-verbal vocalizations: Koeda et al. (2013) examined perception of emotional vocalizations by Canadian and Japanese listeners, Laukka et al. (2013) examined Swedish listeners' perception of vocalizations from four countries, and Sauter (2013) examined the role of motivation in the in-group advantage for emotion recognition by presenting listeners with vocalizations produced by in- or out-group members.

Discussing the similarity between music and speech emotion expression, Juslin (2013) forwarded the argument that this similarity lies at the “core” or basic emotion level, and that more complex emotions are more domain-specific. Several authors empirically tested the similarity and contrasts between music and vocal expression. Margulis (2013) posited that the relative preponderance of repetition in music compared to speech contributes to a fundamental difference between the two domains. Quinto et al. (2013) showed differences in the functions of pitch and rhythm between these domains. Weninger et al. (2013) synthesized information from databases including speech, music, and environmental sounds, and thereby took a step toward a holistic computational model of affect in sound. To aid future cross-domain research, Paquette et al. (2013) presented a new validated set of stimuli—a musical equivalent to vocal affective bursts. Bowling (2013) reviewed the affective character of musical modes, based in the biology of human vocal emotion expression, and Bryant (2013) further argued that research on music and emotion might benefit from research on form and function in non-human animal signals.

Three papers examined developmental and lifespan changes. Corbeil et al. (2013) contrasted the perception of speaking and singing in infancy, and found that it is not the domain (music or speech) that matters but rather the level of (positive) emotion. Wang et al. (2013) examined early auditory deprivation, asking children with cochlear implants to imitate happy and sad utterances. Vieillard and Gilet (2013) found an increase in positive responding to music with aging.

In sum, the main contribution of this Research Topic, along with highlighting the variety of research being done already, is to show the places of contact between the domains of music and vocal expression that occur at the level of emotional communication. In addition, we hope it will encourage future dialog among researchers interested in emotion in fields as diverse as computer science, linguistics, musicology, neuroscience, psychology, speech and hearing sciences, and sociology, who can each contribute knowledge necessary for studying this complex topic.

## Conflict of interest statement

The authors declare that the research was conducted in the absence of any commercial or financial relationships that could be construed as a potential conflict of interest.
